# Language Screening in 3-Year-Olds: Development and Validation of a Feasible and Effective Instrument for Pediatric Primary Care

**DOI:** 10.3389/fped.2021.752141

**Published:** 2021-11-23

**Authors:** Daniel Holzinger, Christoph Weber, William Barbaresi, Christoph Beitel, Johannes Fellinger

**Affiliations:** ^1^Institute of Neurology of Senses and Language, Hospital of St. John of God, Linz, Austria; ^2^Research Institute for Developmental Medicine, Johannes Kepler University Linz, Linz, Austria; ^3^Institute of Linguistics, University of Graz, Graz, Austria; ^4^Department for Inclusive Education, University of Education Upper Austria, Linz, Austria; ^5^Division of Developmental Medicine, Department of Pediatrics, Boston Children's Hospital and Harvard Medical School, Boston, MA, United States; ^6^Division of Social Psychiatry, University Clinic for Psychiatry and Psychotherapy, Medical University of Vienna, Vienna, Austria

**Keywords:** pediatric, feasibility, validity, pre-school age, language disorder, language-delayed children

## Abstract

**Objective:** The study was aimed at evaluating the validity and feasibility of SPES-3 (Sprachentwicklungsscreening), a language screening in 3-year-old children within the constraints of regular preventive medical check-ups.

**Methods:** A four-component screening measure including parental reports on the child's expressive vocabulary and grammar based on the MacArthur Communicative Development Inventory and pediatrician-administered standardized assessments of noun plurals and sentence comprehension was used in a sample of 2,044 consecutively seen children in 30 pediatric offices. One-hundred forty-four children (70 who failed and 74 who passed the screener) comprised the validation sample and also underwent follow-up gold standard assessment. To avoid verification and spectrum bias multiple imputation of missing diagnosis for children who did not undergo gold standard assessment was used. Independent diagnoses by two experts blinded to the screening results were considered gold standard for diagnosing language disorder. Screening accuracy of each of the four subscales was analyzed using receiver operator characteristic (ROC) curves. Feasibility was assessed by use of a questionnaire completed by the pediatricians.

**Results:** The two parental screening subscales demonstrated excellent accuracy with area under the curve (AUC) scores of 0.910 and 0.908 whereas AUC scores were significantly lower for the subscales directly administered by the pediatricians (0.816 and 0.705). A composite score based on both parental screening scales (AUC = 0.946) outperformed single subscales. A cut off of 41.69 on a *T*-scale resulted in about 20% positive screens and showed good sensitivity (0.878) and specificity (0.876). Practicability, acceptability and sustainability of the screening measure were mostly rated as high.

**Conclusion:** The parent-reported subscales of the SPES-3 language screener are a promising screening tool for use in primary pediatric care settings.

## Introduction

Depending on the definition used, 2–10% of pre-school-age children experience delayed language acquisition, which makes language disorder (LD) one of the most prevalent developmental disorders (1, 2). However, there is no generally accepted definition of what constitutes a LD. A recent consensus statement on terminology and criteria for language problems in children (3) has resulted in the endorsement of the term Developmental Language Disorder (DLD) for language difficulties that are associated with functional impairment and poor prognosis but have no known biomedical etiology. DLD continues to be a clinical diagnosis since functional impairment and prognosis need to be assessed by appropriately trained clinicians. In addition, the degree of language delay and the linguistic dimensions (phonology, vocabulary, morphology, syntax, pragmatics) and modalities (expressive and receptive) encompassed have not been specified in the consensus document.

In an English population study, Norbury et al. (2) found a prevalence of DLD (of unknown origin) of 7.58%, while 2.34% of LDs were associated with intellectual disability and/or a medical diagnosis (total approximately 10% of LDs from all causes). They defined DLD as scores of −1.5 standard deviations (SD) and below on at least two of five language domains. Similarly, other researchers (4–6) classified a child as “specifically language impaired” whenever language performance was below −1.25 SD in at least two language domains measured by norm-referenced tests. In the absence of a generally accepted measurable gold standard for the definition of LDs, we based our definition on the previously mentioned classifications, which are commonly used in research, with an expected prevalence rate of about 10%.

LDs can affect multiple domains of development through adolescence and adulthood. Children with delayed language development are at increased risk for poor socio-emotional, health (7), behavior and academic outcomes (8, 9) and later unemployment (10) with corresponding costs and loss of human potential.

There is growing evidence that intervention for children with LD may be effective. Direct treatments by a specialist and indirect treatments mediated by caregivers have been shown to have positive effects (11–14). Hence, it is essential to identify children who require educational or therapeutic support in their language learning in order to offer timely and effective intervention.

Based on a number of methodological problems identified in their systematic review of language screening (e.g., lack of information on the effects of age, setting and administrator on screening accuracy) and insufficient evidence for long-term outcomes of language interventions, Nelson et al. (15) did not recommend universal language screening. In a more recent systematic review, Wallace et al. (16) reported on the accuracy of some screening measures for identification of children with language impairment although evidence of feasibility in primary-care settings remained inadequate. Consequently, the U.S. Preventive Taskforce did not recommend universal screening for language delay (17). Given the international systematic reviews (1, 15) and following the National Health and Medical Research Council (18), the German Institute for Quality and Efficiency in Health Care (19) also considered the available evidence insufficient to recommend the implementation of language screening in Germany. In the same vein, a systematic review evaluating the effectiveness of systematic population-based screening for specific language impairment in pre-school children in Germany (20) concluded that the accuracy of German screening measures had not yet been sufficiently examined.

Systems for general health check-ups have been established in many countries in the Western world and could be suitable opportunities for identification of language delays. However, due to a lack of accurate and feasible instruments, routine well-baby check-ups are generally not used for systematic language screening. One exception is in the Netherlands, where a five-minute interview/test procedure (VTO language screening) administered by a youth health care physician to 24 month-old children led to more cases with language impairment being identified than by the regular procedure (0.4–2.4%), and at a significantly younger age than in regions in which the regular detection procedures were used (21). However, as demonstrated by the low number of cases, many 2-year-old children with language impairment were not identified (low sensitivity of 24–52%). Given the high instability of language development trajectories in young children, Law et al. (1) recommended the age span of 3–5 years as the optimal period for language screening.

A combination of (i) observations by parents with extensive long-time knowledge of their children's behaviors in everyday life and (ii) standardized assessments by pediatricians is essential to avoid assessment bias (22). A comparative study of direct assessments and parent reports of language and pragmatics revealed patterns of difference that indicated a need to collect assessment data from multiple informants (23). Nevertheless, convergent validity of parent-reported tools in comparison to direct assessments of language is usually high (24, 25). Mere elicitation of parental concerns has therefore also been described as a valid method for identifying an increased risk for developmental disorders (26–28).

Particularly at the age of 3 years, grammatical knowledge is a good marker of language development (29–31). Furthermore, grammatical skills can usually be reliably assessed within a shorter time than expressive or receptive vocabulary knowledge. In addition, a comprehensive measure for identifying language disorders must refer to both production and comprehension (4). Another reason for including language reception in a screening measure is its ability to predict the persistence of language difficulties (32). As parental reports of language comprehension have often been found to overestimate children's skills (33), a direct assessment of language comprehension should be considered.

Upper Austria is a federal state with a population of 1.45 million inhabitants (with 13.297 births in 2007). Well-child visits are provided free of charge by community pediatricians and general practitioners. In 2010, when the validation study was conducted, 9,125 health check-ups (68.6% of the children born in 2007) were carried out with 3-year-old children, 66% of which by pediatricians and the remainder by general practitioners.

The aim of this study was the validation of a screening tool for LDs for 3-year-old German-speaking children in a pediatric primary-care setting in Upper Austria. The new instrument, the SPES-3 (Sprachentwicklungsscreening) that has been developed in a pilot study includes both parent observations and direct assessments of the child by the primary-care pediatrician. As required for comprehensive language assessments, the screening measure takes various linguistic domains (grammar and vocabulary) and modes (receptive and expressive) into account. For use in primary-care settings, the screening tool must have high acceptability and require little time to administer while maximizing accuracy.

## Methods and Procedures

### Construction of the Screening Measure and Pilot Testing

Both parent-administered screening scales are based on the MacArthur-Bates Communicative Development Inventories (MCDI), Level III (34), and require parents to systematically report their observations of their child's development of expressive grammar and expressive vocabulary. Inspired by the MCDI concept for grammar assessment a number of morphosyntactic structures of German that typically emerge around age 3 were selected to be presented to the child in the form of 27 pairs of correct and incorrect options. Parents are asked to select the option they are more likely to observe in their child's spontaneous use of language. For expressive vocabulary, 100 words from the MCDI-III English word list (34) translated into Austrian German (35) were chosen. Parents are asked to indicate whether their child uses the words in their expressive communication or not.

The screening subscales administered by the pediatricians were compiled from pre-existing subtests of a German standardized language test (Sprachentwicklungstest SETK 3-5; (36), The first subscale includes 20 items that assess the production of noun plurals: The pediatrician presents and names a pictured item in the singular and asks the child to produce the respective plural form supported by a picture that shows several identical items. The second subscale assesses sentence comprehension: Single sentences are read aloud (9 items), and the child is asked to point to the corresponding picture from a selection of four. For an overview of all screening scales (see Table 1).

**Table 1:** Screening subscales.

	**Parent report**	**Pediatric assessment**
Vocabulary expressive	Expressive vocabulary (MCDI-3 word list, Austrian version)	
Grammar expressive	Expressive grammar (inspired by MCDI-3)	Noun plurals (SETK 3-5)
Grammar receptive		Sentence comprehension (SETK 3-5)

*MCDI-3, MacArthur-Bates Communicative Development Inventories Level III; SETK 3-5, Sprachentwicklungstest (language test for age 3–5 years)*.

The preliminary version of the screening procedure was first used in 2006 in a pilot study in close cooperation with the Pediatric Association and the Department of Health and Social Affairs in Upper Austria, with the aim of developing language screening instruments to be used in pediatric primary care within regular health check-ups at ages 2 and 3. The pilot study included 1,730 non-preselected 3-year-old children with German as their first language, who were consecutively assessed by a group of 24 primary care pediatricians recruited from across the state of Upper Austria (whose participation was voluntary).

Based on the pilot study, the *parental report of expressive grammar* scale, which initially contained 27 items, was shortened by excluding 14 items with low response rates, low difficulty and low item-scale correlation. Thus, the final screening measure used in the validation study covered 13 items for expressive grammar, 100 items for expressive vocabulary, 20 items for noun plurals and 9 items for sentence comprehension. All items were scored either as 0 (does not apply/false) or 1 (applies/correct), and for each subscale a sum over the item scores was computed. Internal consistency (Kuder and Richardson Formula 20) was excellent for the parental screening scales (ρ_KR20_ = 0.98 for expressive vocabulary and 0.90 for expressive grammar) and adequate for the screening scales directly administered with the children [ρ_KR20_ = 0.78 for noun plurals and sentence comprehension; (36)] For the questionnaires to be completed by the parents (expressive grammar and expressive vocabulary) cut-off scores for the validation study were derived from the 10th percentiles (SD 1.25) based on the final instruments, as suggested by the literature (4–6). Normative data to determine cut-off scores were available for both of the standardized assessments to be completed by pediatricians (noun plurals and sentence comprehension).

### Gold Standard Assessment of Language Disorder

Given the lack of well-defined standards for the diagnosis of LDs, independent expert diagnoses by two experienced clinical linguists blinded to the language-screening results were considered the gold standard. Their diagnostic decisions on LD were based on the results of two standardized tests assessing the linguistic domains of expressive sentence grammar, noun plural production, sentence comprehension [SETK 3-5; (36)] and expressive vocabulary [AWST-R; (37)] that had been administered by other linguists and a short video sample (5–10 min) of spontaneous language of each child in a play and/or dialogic picture-book situation. For their decision on LD both clinical linguists followed international research (4–6) and classified a child as language delayed when language performance was at about the 10th percentile or lower in at least two of the four measured language domains and observations of spontaneous language production confirmed the significant language difficulties. Inter-rater reliability (kappa) between the linguists' diagnoses (+/– LD) was 0.95. Discrepancies were resolved by consensus decisions between the two raters.

For the assessment of non-verbal intelligence, the Snijders Oomen Non-verbal Intelligence Test [SON-R 2 ½-7; (38)] was administered by clinical psychologists. Pure tone audiometry was used to assess hearing.

### Feasibility Measures

Feasibility was measured primarily by use of a questionnaire completed by the pediatricians who participated in the study and by the completeness of screening tests administered. Following the guidelines suggested by Bowen et al. (39), four dimensions of feasibility were investigated. All questions of the pediatric questionnaire were rated on a 4-point Likert scale (very good-good-difficult-very difficult).

#### Practicality

Practicality was operationalized by the extent to which administration of the screening was considered possible within the time constraints of pediatric primary care. In addition, the pediatricians were asked about the ease of administration of each of the two screening measures (noun plurals and sentence comprehension) and to evaluate parental difficulties in completing the two parental subscales of the screening. In addition, the pediatricians ranked five pre-specified factors that might challenge the completion of the screening measure within their respective settings.

#### Acceptability

Acceptability refers to the children's, parents' and pediatricians' reactions to the screening measures. Child acceptance was measured by the percentage of screening subscales administered by the pediatricians that were fully completed, as this reflects child compliance with the test. In addition, the pediatricians assessed parental acceptance of the inclusion of language screening in the 3-year medical check-up. Finally, the pediatricians were asked to rate the meaningfulness of including a language screening within the regular well-baby check-ups.

#### Sustainability

Sustainability refers to the likelihood of language screening in this form being continued within the present system of preventive medical care in Austria. Pediatricians were asked in the questionnaire whether they intended to continue the language screening after the study ended (yes, to a limited extent, no).

### Study Procedures and Recruitment

In 2009, all primary care pediatricians of the province of Upper Austria were invited to participate in the validation study of the language screening. Thirty-six out of 60 pediatricians participated in a half-day training and in the subsequent implementation of the screening procedures. The participating pediatricians served all major geographical areas of Upper Austria. Over a 1-year period (2010) 2,635 3-year-old children (19.8% of the entire 2007 Upper Austrian birth cohort) were screened by the 30 pediatricians who ultimately participated in the study.

Overall, 591 children were excluded from the study: 31 children with missing data on their date of birth, one child with missing data on the screening date, 95 children who were outside the age range (34–38 months), 349 children from families whose primary family language was other than German, 95 children with missing data on both parental screening subscales and 22 with missing data on both pediatric screening subscales. The remaining sample (*n* = 2,044) equaled 15.4% of the entire 2007 birth cohort and about 22% of the children born in 2007 by native-born mothers. The mean age was 36.03 months (SD = 0.994). 50.9% were boys, 4.9% were multiple births, 50.7% of the children had older siblings, and 8.6% of children were born prematurely. Compared to the 2007 birth cohort for Upper Austria, the sample was representative in terms of sex ratio and prematurity rate. Multiple births were slightly overrepresented (4.9% vs. 3.4% in the birth cohort; χ^2^(1) = 14.003, *p* < 0.001). To assess the representativeness of the sample in terms of maternal education (proportion of mothers with university entrance qualification or tertiary degree), we calculated an age-adjusted comparison value based on the educational level of all women (i.e., not only mothers) from Upper Austria. Among the participating families the percentage of mothers with either a university entrance qualification or a tertiary degree was comparable to the age-adjusted population (39.4% vs. 41.6%; χ^2^(1) = 4.072, *p* < 0.05).

The full screening was administered in the course of well-child visits in the pediatric medical practices. Parents completed a screening package that included demographic information in addition to their language observations (expressive grammar and expressive vocabulary). All parents gave written permission for scientific use of their children's anonymized data for scientific purposes. The study was approved by the ethics committee of the Hospital of St. John of God, Linz. Pediatricians administered two screening subscales (noun plurals and sentence comprehension). All screening data were sent back to the clinic conducting the research. In accordance with Tomblin et al. (40), a screening test was considered a fail if the results of at least two of the four subtests were 1.25 standard deviations below the age norm. Using this ex-ante definition for positive screening results, 21.7% of the sample was considered a screening fail.

**Figure 1:**
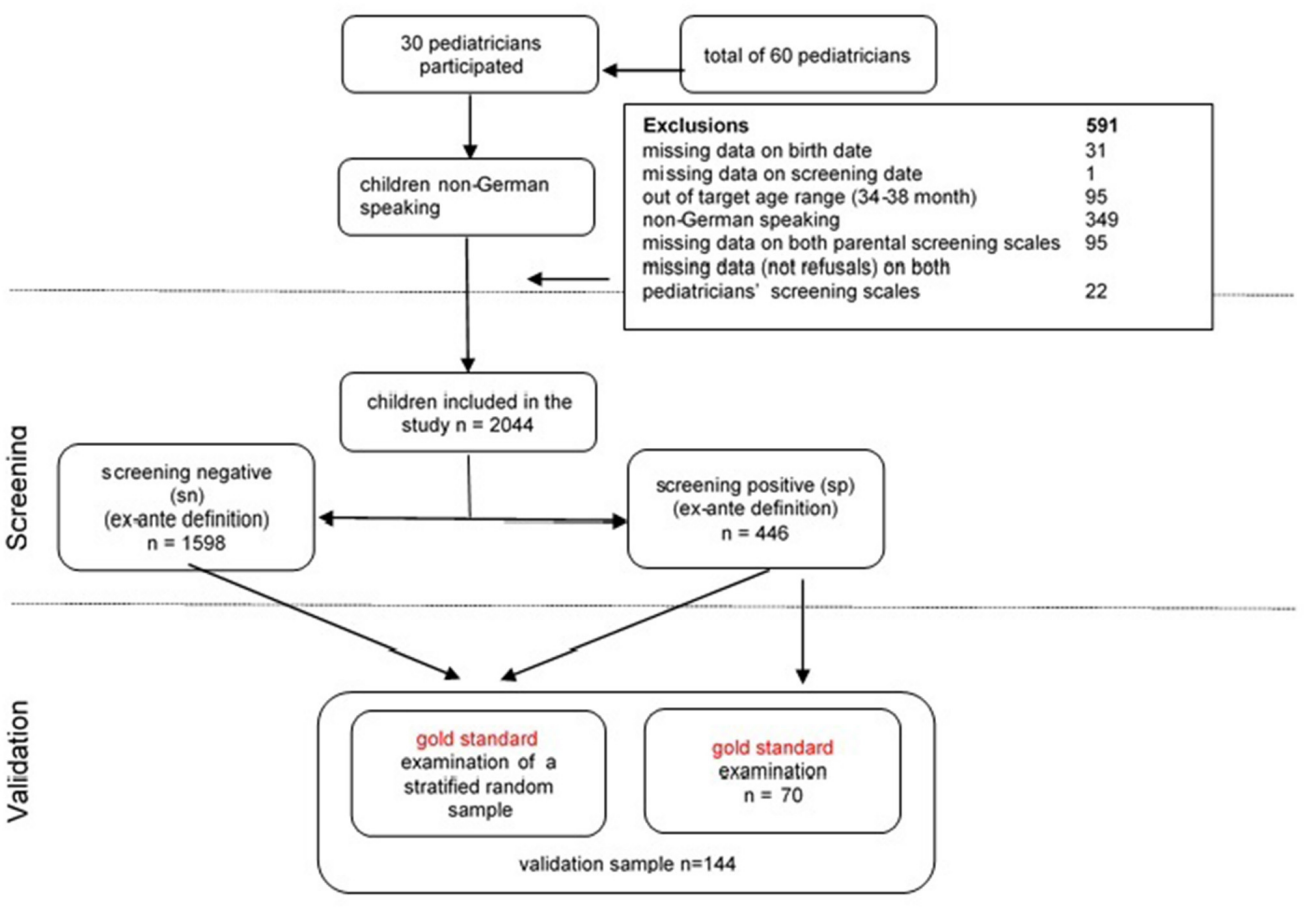
Recruitment and participation.

To validate the screening measures, a sample for full gold standard assessment was recruited in a two-step procedure. All pediatricians were instructed to refer children with positive screening results to a single specialized program for gold standard examination, which was performed in 70 children. Second, to evaluate specificity of the screening tool, four pediatricians from different regions were asked to invite a random subsample of children from the whole cohort, excluding those (*n* = 70) who had already undergone the gold standard assessment. The sample was stratified by gender, maternal education, position among siblings and single parenthood. The recruitment procedures (see Figure 1) led in total to a validation sample of *n* = 144 (i.e., 7% of the total sample), that did not deviate from the remaining sample in terms of demographic variables (see sample description).

### Analytic Strategy

First, we report descriptive statistics for the total sample and the validation sample. Second, we present results on the screening accuracy of each subscale based on receiver operator characteristic (ROC) analyses. Areas under the curve (AUCs) ≥0.9 are regarded as excellent, AUCs ≥0.8 and < 0.9 as good, AUCs ≥0.7 and < 0.8 as fair, and tests with AUCs < 0.7 as poor (41). DeLong's method for comparing AUCs of different tests (42) was applied to analyze differences in diagnostic accuracy between the subtests. Further, logistic regression was applied to identify subscales with significant independent contributions to the prediction of the gold standard diagnosis. The aim of this analytic step was to reduce the number of screening subtests while optimizing diagnostic accuracy. Lastly, we evaluated possible cut-off scores for the final screening composite by estimating sensitivity, specificity, positive predictive values (PPV), negative predictive values (NPV), and diagnostic likelihood ratios for positive and negative screening results (DLR+ and DLR–, respectively). DLR+ and DLR– are alternative measures of the accuracy of a diagnostic test and have the advantage, unlike predictive values, not to depend on the prevalence of the disorder under investigation [(43); for an explanation see Supplementary Material]. DLR+ is the multiplicative change in the pre-screening odds of having a LD given a positive screening result (i.e., post-screening odds = DLR+ × pre-screening odds) and DLR– is the change in the pre-screening odds of having a LD given a negative screening result (post-screening odds = DLR– × pre-screening odds). Following Jaeschke et al. (44), DLR+ values ≥ 10 and DLR– ≤ 0.1 indicate large changes in pre-screening odds, DLR+ ≤ 10 and >5, and DLR– > 0.1 and ≤ 0.2 indicate moderate changes, DLR+ ≤ 5 and >2, and DLR– > 0.2 and ≤ 0.5 indicate small changes. DLR+ <2 and DLR– > 0.5 are rarely important. Logistic regression models were conducted using M*plus* 8 (45), for the ROC analysis the pROC package (46) in R was employed, and cut-off values were determined with the R-OptimalCutpoints package (47).

Notably, the recruitment procedure led to oversampling of children with positive screening results, which is not uncommon for screening validation studies (48). Ideally, the validation sample should reflect the patient population, the given overrepresentation of positive screening results induces bias in measures of screening accuracy [i.e., verification bias; (49)]. In order to deal with this bias, we used multiple imputation (MI) of missing diagnosis status for children who did not undergo gold standard assessment (50). Missing values on screening subscales (ranging between 0.4 and 12.8% in the full sample) were also imputed. We used the Blimp imputation software (51, 52) to generate 50 imputed data sets using a chained equation imputation procedure that takes the clustering of children within pediatricians into account. Beside the study variables (i.e., sociodemographic variables and screening results) we used various auxiliar variables (e.g., parental concern about language development) that were predictive of diagnosis status. Estimates and their standard errors were computed according to Rubin's combining rules (53). Even though recent simulation studies do not indicate that high proportions (up to 90%) of missing data might bias estimates based on MI data sets (54), we also report results for the original validation sample (*n* = 144) as Supplementary Material for completeness.

## Results

**Figure 2:**
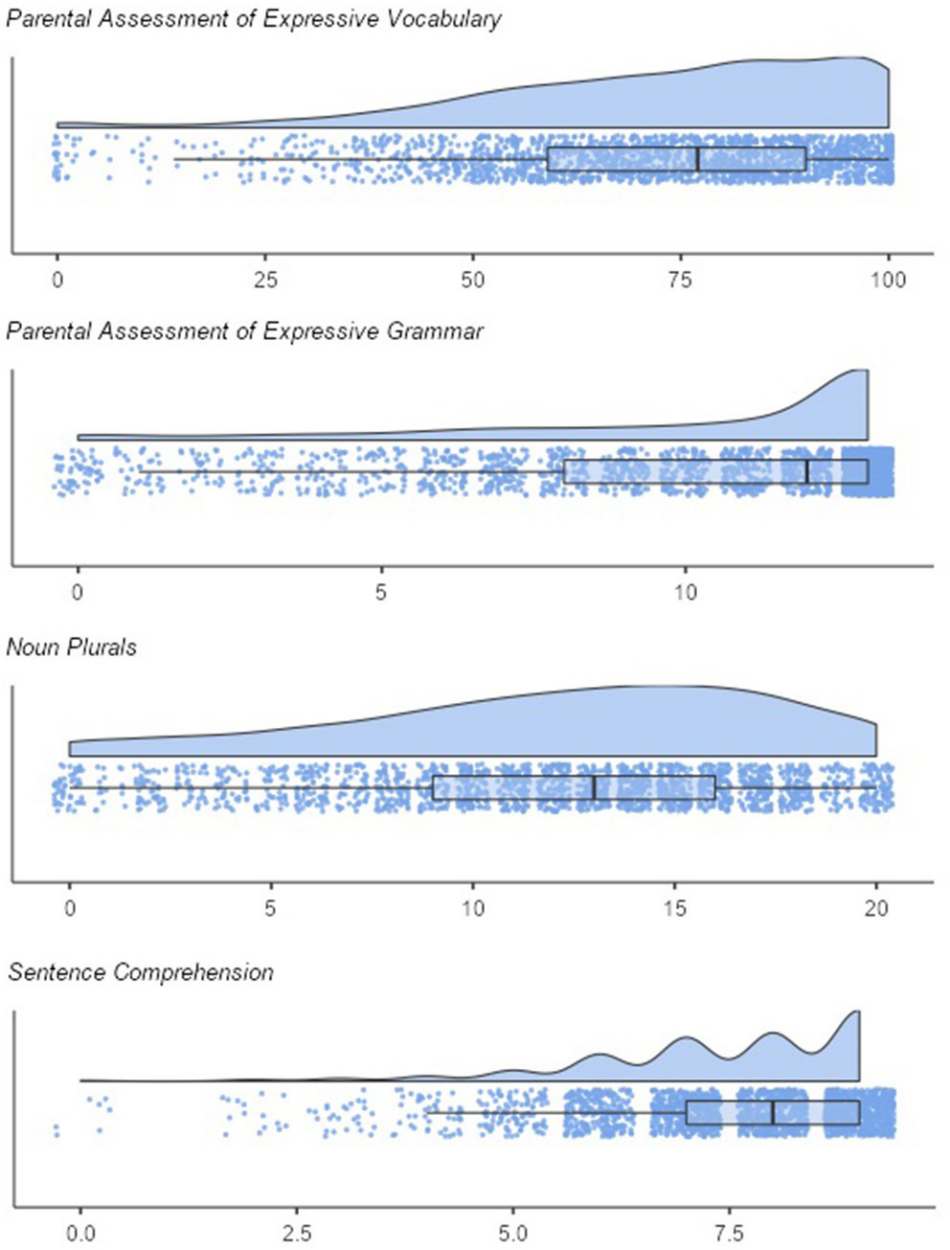
Distribution of the screening subscales (raw scores).

Figure 2 shows the distributions of the screening subscales. As expected, the scores on all scales were skewed to the left. Means and standard deviations were *M* = 10.22 (SD = 3.67) for expressive grammar and *M* = 73.00 (SD = 21.76) for expressive vocabulary, *M* = 12.05 (SD = 5.19) for noun plurals, *M* = 7.48 (SD = 1.48) for sentence comprehension. The correlations between screening subscales were moderate to high. The parental scales correlated with *r* = 0.56 (*p* < 0.001). The correlation of the pediatricians' scales was *r* = 0.40 (*p* < 0.001). Further correlations were: *r*_expressive vocabulary, sentence comprehension_ = 0.30, *p* < 0.001; *r*_expressive vocabulary, noun plurals_ = 0.40, *p* < 0.001; *r*_expressive grammar, sentence comprehension_ = 0.36; *p* < 0.001; *r*_expressive grammar, noun plurals_ = 0.43; *p* < 0.001.

Moreover, analyses showed that children with positive screening results (ex-ante definition) who attended the gold standard assessment showed lower scores in three subscales than children with positive screening results who did not attend the assessment (expressive grammar: *d* = 0.60, *p* < 0.001; sentence comprehension: *d* = 0.29, *p* < 0.05; noun plurals: *d* = 0.51; *p* < 0.001). Thus, the validation sample may also be subject to spectrum bias (i.e., screening positives include primarily the “sickest of the sick” and not the full spectrum of positive screens), which is associated with overestimation of sensitivity, specificity, and AUC (49). However, the used multiple imputation procedure is also suitable to counteract spectrum bias. Based on the gold standard, 27.8% of the validation sample (*n* = 144) had a LD. Notably, all children with LD had positive screening results (i.e., sensitivity = 1.00). After imputation 11.7% of the children are classified as having a LD.

As children are clustered within pediatricians, we estimated the intraclass correlation (ICC) to evaluate whether there are differences between pediatricians in the subscales. We found that pediatricians accounted for ≈14% of the differences in the pediatrician-reported subscales (ICC_noun plurals_ = 0.144, *p* < 0.001; ICC_sentence comprehension_ = 0.139, *p* < 0.001). If differences between pediatricians were due to population differences in the catchment areas, we would also expect comparable ICCs for the parent reports. However, ICCs for parent reports were smaller (ICC_expressive vocabulary_ = 0.049, *p* = 0.005, ICC_expressive grammar_ = 0.021, *p* = 0.05). Thus, these findings indicate that pediatricians differed significantly in their application of the screening tools, which calls into question the objectivity of implementation.

### Diagnostic Accuracy of Subscales

Results of the ROC analysis for the screening subscales are shown in Table 2. The AUCs ranged from fair (AUC_sentence comprehension_ = 0.705, DeLong 95% CI = [0.623, 0.786]) to excellent (AUC_expressive grammar_ = 0.910, DeLong 95% CI = [0.859, 0.960]). The second parent-reported subscale showed an almost identical excellent AUC value (AUC_expressive vocabulary_ = 0.908, DeLong 95% CI = [0.864, 0.952]). The AUC for noun plurals was good (AUC_noun plurals_ = 0.816, DeLong 95% CI = [0.745, 0.887]). As indicated by DeLong tests for paired ROC curves, parent-reported scales outperformed the screening subscales administered by pediatricians. All AUC differences between parent-reported and pediatrician-administered scales were significant, and noun plurals outperformed sentence comprehension.

**Table 2:** Diagnostic accuracy of the screening subscales.

	**Language disorder**							
	**Yes**	**No**				**AUC-differences—** ***t*** **-values (DeLong)**		
	***M*** **(SD)**	***M*** **(SD)**	***r*** **_pb_**	**AUC**	**95%-CI**	**Expressive** **Vocabulary**	**Expressive** **grammar**	**Sentence** **comprehension**
*N* = 2,044								
Expressive vocabulary	33.656 (17.563)	70.810 (19.864)	−0.515***	0.908	(0.864; 0.952)			
Expressive grammar	1.851 (2.906)	9.165 (4.103)	−0.556***	0.910	(0.859; 0.960)	−0.039		
Sentence comprehension	5.462 (2.388)	7.026 (1.734)	−0.251***	0.705	(0.623; 0.786)	4.711***	5.804***	
Noun plurals	3.426 (4.492)	10.056 (5.903)	−0.363***	0.816	(0.745; 0.887)	2.440*	2.629*	−2.848**

* *p < 0.05*;

** *p < 0.01*;

*** *p < 0.001. r_pb_, point biserial correlation*.

To examine independent contributions of subscales to predicting the gold-standard diagnosis logistic regression analyses were performed (Table 3). The two parent-reported subscales independently predicted LD. After controlling for parent-reported screening tests, none of the pediatrician-administered subscales significantly predicted LD. Standardized coefficient (b) for the expressive vocabulary subscale was −0.408 (*p* < 0.001) and −0.388 (*p* < 0.001) for the expressive grammar subscale. Notably, as indicated by the overlapping confidence intervals of the standardized logistic regression coefficients, both parent reported scales had roughly the same weight in predicting LD.

**Table 3:** Logistic regression predicting LD on the basis of screening subtests.

	***b*** **(SE)**	**Stand. ***b*** [95%-CI]**	**OR**
Expressive vocabulary	−0.057*** (0.015)	−0.408 [−0.579, −0.237]	0.945
Expressive grammar	−0.315*** (0.066)	−0.388 [−0.536, −0.241]	0.730
Sentence comprehension	0.052 (0.153)	0.028 [−0.132, 0.188]	1.053
Noun plurals	−0.092 (0.058)	−0.160 [−0.351, 0.031]	0.912
Threshold	−3.917		
*R* ^2^		0.634***	

*OR, odds ratio*;

*** *p < 0.001*.

### Diagnostic Accuracy of the Composite Screening Score

Given the results of the logistic regression models, a composite screening score based on both significant predictors (expressive vocabulary and expressive grammar) was computed. As both parent reported scales contributed almost equally to the prediction of LD, a composite score was computed as the mean of the z-scores of expressive vocabulary and expressive grammar. The AUC for the composite score was excellent at 0.946 (DeLong 95% CI = [0.883, 1.000]). DeLong tests for paired ROC curves indicate that the composite outperformed the single parent reported scales (composite score vs. expressive vocabulary: ΔAUC = 0.038, *t*-value = 2.380, *p* = 0.019; composite score vs. expressive grammar: ΔAUC = 0.036, *t*-value = 2.102, *p* =0.037).

### Cut-Off Estimation

In the next step, cut-off values for the screening composite were estimated (Table 4). For ease of interpretation, the composite score was transformed into a *T* metric (i.e., *M* = 50, SD = 10). First, we estimated the cut-off by setting sensitivity equal to specificity using the “SpEqualSe” criterion in the Optimal Cutoff Package (47). A cut-off at 41.69 was most efficient. Table 5 reports the classification results for this cut-off that resulted in satisfactory accuracy statistics: sensitivity = 0.878 (95%-CI = [0.770, 0.985]), specificity = 0.876 (95%-CI = [0.856, 0.895]), PPV = 0.438 (95%-CI = [0.333, 0.544]), NPV = 0.984 (95%-CI = [0.967, 1.000]), DLR+ = 7.078 (95%-CI = [5.779, 8.378]), DLR– = 0.140 (95%-CI = [0.018, 0.261]). The cut-off would have resulted in 20.0% screening fails and consequently in a relatively high number of clinical evaluations required. Lower cut-offs resulted in fewer screening fails and higher PPV, DLR+, DLR– and specificity, but also in lower sensitivity (see Table 4). Thus, lower cut-offs would yield more false-negative results.

**Table 4:** Diagnostic accuracy statistics for various cut-offs.

**Cutoff**	**%-Screening positives**	**Sensitivity**	**Specificity**	**PPV**	**NPV**	**DLR+**	**DLR-**
35	0.092	0.609 (0.475, 0.742)	0.965 (0.954, 0.977)	0.658 (0.538, 0.779)	0.956 (0.929, 0.983)	17.813 (10.418, 25.187)	0.406 (0.268, 0.543)
36	0.111	0.661 (0.532, 0.790)	0.950 (0.936, 0.963)	0.591 (0.470, 0.713)	0.961 (0.936, 0.986)	13.294 (8.735, 17.843)	0.357 (0.211, 0.493)
37	0.124	0.697 (0.568, 0.828)	0.939 (0.924, 0.954)	0.557 (0.438, 0.677)	0.965 (0.941, 0.988)	11.518 (7.936, 15.100)	0.323 (0.187, 0.460)
38	0.138	0.742 (0.615, 0.868)	0.929 (0.913, 0.945)	0.536 (0.0.418, 0.654)	0.969 (0.947, 0.992(	10.547 (7.545, 0.13.548)	0.278 (0.143, 0.413)
39	0.150	0.779 (0.655, 0.903)	0.919 (0.902, 0.936)	0.516 (0.399, 0.632)	0.973 (0.952, 0.995)	9.704 (7.156, 12.252)	0.240 (0.106, 0.375)
40	0.169	0.820 (0.701, 0.939)	0.904 (0.886, 0.922)	0.485 (0.374, 0.596)	0.978 (0.958, 0.997)	8.553 (6.620, 0.10.480)	0.199 (0.068, 0.330)
41	0.189	0.865 (0.755, 0.976)	0.886 (0.867, 0.905)	0.456 (0.348, 0.564)	0.983 (0.965, 1.000)	7.612 (6.127, 9.907)	0.152 (0.028, 0.276)
42	0.202	0.879 (0.773, 0.985)	0.873 (0.853, 0.892)	0.433 (0.328, 0.538)	0.984 (0.967, 1.000)	6.918 (5.678, 8.159)	0.139 (0.018, 0.259)

*95%-CI in brackets*.

**Table 5:** Classification table.

	**Language disorder**	**Language disorder**	**Total**
	**No**	**Yes**	
Screening pass	1,609.90	26.18	1,636.08
Screening fail	229.25	178.67	407.92
Total	1,839.15	204.85	2,044

*Decimal absolute values are presented, because results are based on the average across 50 imputed data sets*.

### Feasibility

Completed questionnaires that addressed feasibility were returned by 23 (77%) out of the 30 pediatricians participating in the study.

#### Practicality

According to the pediatricians, 13.6% of the parents considered completion of the questionnaire to be “difficult”. However, the remainder rated it most often as “very easy” (47.8%) or “easy” (34.8%). Practicality of the screening measures within the time limits of preventive medical care was rated mostly as good (69.6%) or very good (8.7%). In 21.7% of the cases, it was considered “difficult”, but never “very difficult”. Ease of administration of the sentence comprehension screening scale was assessed as significantly higher than that for noun plurals (expressive grammar). Ease of administration of the receptive measure was rated in most cases as good (60.9%) and very good (34.8%), and as difficult by only one pediatrician (4.3%). In contrast, 47.8% rated the screening of noun plurals as difficult, 39.1% as good and only three pediatricians (13.0%) as very good. Among the factors that might complicate or even prevent administration of language screening, lack of or insufficient follow-up was ranked highest (40%), followed equally by insufficient training of pediatricians (20%) and insufficient funding (20%), and then by limited meaningfulness of language screening (13.3%). Only one respondent cited time constraints (4.3%).

#### Acceptability

General parental acceptance of language screening included in preventive health care was rated as “very good” by 43.5% and as “good” by all remaining pediatricians (52.2%). The percentage of children refusing their cooperation in the pediatrician's administration of the assessment of noun plurals was 12.8%, and for the assessment of sentence comprehension 5.5%, indicating satisfying acceptance of the assessment of language reception by the children, although child participation is no longer required for the final screening package that is exclusively based on parent report. A great majority of pediatricians rated the meaningfulness of language screening within the regular medical checkups as very good, another three (13.9%) as good, and only 2 expressed concerns (9.1% “difficult”).

#### Sustainability

Most pediatricians (82.6%) reported that they would continue the SPES-3 language screening beyond the study, while the remainder would stop. It is noteworthy that no specific additional funding for language screening could be provided.

## Discussion

This study evaluated the diagnostic accuracy and feasibility of a newly developed language screening measure (SPES-3) for children aged 3 years in pediatric primary care within regular well-baby check-ups. In addition to the implementation by community pediatricians in consecutively evaluated children, the extensive sample size and the use of multiple imputation to avoid verification and spectrum bias are strengths of this study. The two parent-reported subscales (expressive grammar and vocabulary development) showed excellent accuracy (AUC.910, 0.908). Although AUC scores for pediatric subscales were significantly lower, they were good for the noun-plural subscale (0.816) and fair for the sentence comprehension subscale (0.705). Nevertheless, logistic regression analysis showed that none of the pediatrician-administered subscales significantly increased the diagnostic accuracy achieved by the parent scales. A composite based on both parent reports showed excellent accuracy (AUC = 0.946) and outperformed the single parental subscales. Our findings are consistent with those of a systematic review on the predictive validity of preschool screening tools for language by Sim et al. (55). Their results exhibited higher sensitivity, specificity and negative predictive value of parent-report screening tools as compared to direct child assessment. This finding supports the richness of parental information that is based on long-time observation of their child's language use in a variety of everyday situations. In contrast, the quality of screening tools based on direct assessments is influenced by the brevity required for use in the regular pediatric office and might be influenced by the relationship between child and examiner. The higher intraclass correlations of the screening tests administered by the pediatricians compared to the parent-reported screening tests may, at least partly, reflect differences in building rapport with the child.

Since screening duration in community settings is critical for their large-scale implementation, stronger validity of parent-reported screening in place of direct assessment by a pediatrician can be viewed as a positive finding in terms of feasibility of the screening tool. Practicability of the new screening measure was assessed to be high, even when direct assessments were included in the screening measure. Pediatricians also rated the acceptability by parents and children as high, and the majority of them regarded the inclusion of a language screening measure within their regular well baby check-ups was meaningful.

By use of ROC analysis a cut off of 41.69 is optimal when equal values for sensitivity and specificity are desired. Predictive values, which depend on the prevalence of the disorder, were NPV = 0.984 and PPV = 0.438. Moreover, we evaluated a broader range of cut-offs. As illustrated by Table 4, there is a trade-off between sensitivity and specificity. The more sensitive is a cut off value, the less specific it is. Due to increasing risk of persistence of language difficulties by increasing age and for reasons of practicability we suggest the cut-off of 41 for this new language screening measure for 3-year-olds to reduce the number of missed children with delayed language development. The relatively low PPV would lead to overreferrals but it must be interpreted with regard to the relatively low rate of diagnoses of LD (11.7%). As language difficulties have a dimensional rather than categorical character, children with false-positive scores in language skills have been shown to perform significantly lower on diagnostic measures than children with true-negative scores (56). Since children “overreferred” for diagnostic testing perform lower on language, they are very likely to also carry more psycho-social and cognitive risk factors associated with language delay. Therefore, diagnostic testing should not be regarded as an unnecessary inconvenience to the family and expense to society, but as an opportunity to identify children with unmet needs and the interventions required to improve their language and social and academic learning even though they are probably less severely affected than children with LD.

Even though direct assessments by pediatricians did not contribute to the predictive quality of the screening measure its administration within existing systems for general health check-ups might still be considered. Medical reports and recommendations are often highly valued by families. In case of screening fails (parent reported scales) a medical professional may still administer a brief assessment of language comprehension (sentence comprehension subscale), that was very well accepted by the children. A delay in language comprehension can be indicative of a more severe and persisting language problem (3) and may be associated with other more general developmental problems (e.g., general developmental delay, autism spectrum disorder, or hearing loss) and thus require follow-up at a multi-professional diagnostic center. Notably, among the possible barriers to universal language screening, lack of insufficient follow-up was ranked highest by the pediatricians who participated in the study, followed by insufficient training of pediatricians and lack of funding. Screening guided tiered referral pathways to local speech-language therapists and/or specialized multi-professional diagnostic centers (for the smaller number of children with complex needs) might be a cost-efficient approach.

The limited size of the original validation sample, the high proportion of screening fails in the validation sample and a more severe character of the language delay as compared to those without clinical follow-up are limitations of this study. However, multiple imputation of missing diagnosis for children without gold standard assessment was used to deal with these biases. Another limitation is the lack of baseline data on other developmental disorders. Whereas, we have provided data on the representativity of the study sample, we do not have information on differences between participating and non-participating pediatricians.

## Conclusion

Parent-reported screening measures for expressive vocabulary and grammar (SPES-3) administered within regular well-baby check-ups in pediatric primary care have been found to be accurate in identifying LDs in 3-year-olds. Administration of screening subscales for noun-plurals and sentence comprehension by pediatricians showed lower specificity and sensitivity and, when added to the parental assessments, did not improve overall accuracy of the screening package. Feasibility within regular preventive check-ups was rated as mostly good or very good by the pediatricians. Ease of administration and acceptance by parents and pediatricians demonstrated in implementation with a large cohort of non-preselected children that the SPES-3 screening measure is valuable and can be recommended for universal language screening at age three in pediatric primary care.

## Data Availability Statement

The datasets presented in this article are not readily available because parents of the children participating in the study have not given their consent to make the data publicly available. Requests to access the datasets should be directed to daniel.holzinger@bblinz.at.

## Ethics Statement

The studies involving human participants were reviewed and approved by Ethikkommission Konventhospital Barmherzige Brüder Linz. Written informed consent to participate in this study was provided by the participants' legal guardian/next of kin.

## Author Contributions

DH and JF: conceptualization. DH, JF, CW, WB, and CB: methodology. CW and CB: formal analysis. DH, JF, and CB: investigation. DH and CW: data curation. DH, JF, WB, and CW: writing—original draft preparation and writing—review and editing. DH: project administration. All authors have read and agreed to the published version of the manuscript.

## Funding

The development of the SPES-3 screener was financially supported by the Department of Health and Social Affairs Upper Austria. Article Processing Charge is funded by the Johannes Kepler University Open Access Publishing Fund.

## Conflict of Interest

The authors declare that the research was conducted in the absence of any commercial or financial relationships that could be construed as a potential conflict of interest.

## Publisher's Note

All claims expressed in this article are solely those of the authors and do not necessarily represent those of their affiliated organizations, or those of the publisher, the editors and the reviewers. Any product that may be evaluated in this article, or claim that may be made by its manufacturer, is not guaranteed or endorsed by the publisher.

## References

[1] Law J Boyle J Harris F Harkness A. Prevalence and natural history of primary speech and language delay: findings from a systematic review of the literature. Int J Lang Commun Disord. (2000) 35:165–88. 10.1080/136828200247133 10912250

[2] Norbury CF Vamvakas G Gooch D Baird G Charman T Simonoff E. Language growth in children with heterogeneous language disorders: a population study. J Child Psychol Psychiatry. (2017) 58:1092–105. 10.1111/jcpp.12793 28921543 PMC5639364

[3] Bishop DV Snowling MJ Thompson PA Greenhalgh T CATALISE consortium (2016). CATALISE: a multinational and multidisciplinary Delphi Consensus Study. Identifying language impairments in children. PloS One 11:e0158753. 10.1371/journal.pone.0158753 27392128 PMC4938414

[4] Tomblin JB Records NL Buckwalter P Zhang X Smith E. Prevalence of specific language impairment in kindergarten children. J Speech Lang Hear Res. (1997) 40:1245–60. 10.1044/jslhr.4006.1245 9430746 PMC5075245

[5] Leonard LB. Children with specific language impairment and their contribution to the study of language development. J Child Lang. (2014) 41:38–47. 10.1017./S0305000914000130 25023495 PMC4429873

[6] Thordardottir E. 12. Proposed diagnostic procedures for use in bilingual and cross-linguistic contexts. In: Armon-Lotem S, de Jong J, Meir N, editors. Assessing Multilingual Children. (2015). pp. 331–58. Bristol, Blue Ridge Summit: Multilingual Matters. 10.21832/9781783093137-014

[7] Clegg J Hollis C Mawhood L. Developmental language disorders—a follow-up in later adult life. Cognitive, language and psychosocial outcomes. J Child Psychol Psychiatry Allied Discip. (2005) 46:128–49. 10.1111/j.1469-7610.2004.00342.x 15679523

[8] Lindsay G Dockrell JE Strand S. Longitudinal patterns of behaviour problems in children with specific speech and language difficulties: child and contextual factors. Brit J Educ Psychol. (2007) 77:811–28. 10.1348./000709906X171127 17173708

[9] Catts HW Fey ME Tomblin JB. A longitudinal investigation of reading outcomes in children with language impairments. J Speech Lang Hear Res. (2002) 45:1142–57. 10.1044/1092-4388(2002/093) 12546484

[10] Law J Rush R Schoon I. Modeling developmental language difficulties from school entry into adulthood: literacy, mental health, and employment outcomes. J Speech Lang Hear Res. (2009) 52:1401–16. 10.1044/1092-4388(2009/08-0142) 19951922

[11] Buschmann A Jooss B Rupp A Feldhusen F Pietz J. Parent based language intervention for 2-year-old children with specific expressive language delay: a randomised controlled trial. Arch Dis Child. (2009) 94:110–6. 10.1136/adc.2008.141572 18703544 PMC2614563

[12] Law J Garrett Z Nye C. The efficacy of treatment for children with developmental speech and language delay/disorder: a meta-analysis. J Speech Lang Hear Res. (2004). 47:924–43. 10.1044/1092-4388(2004/069) 15324296

[13] Roberts MY Kaiser AP. The effectiveness of parent-implemented language interventions: a meta-analysis. Am J Speech Lang Pathol. (2011) 20:180–99. 10.1044/1058-0360(2011/10-0055) 21478280

[14] Roberts MY Kaiser AP. Early intervention for toddlers with language delays: a randomized controlled trial. Pediatrics. (2015) 135:686–93. 10.1542/peds.2014-2134 25733749 PMC4379460

[15] Nelson HD Nygren P Walker M. Screening for speech and language delay in preschool children: systematic evidence review for the US Preventive Services Task Force. Pediatrics. (2006) 117:e298–319. 10.1542/peds.2005-1467 16452337

[16] Wallace IF Berkman ND Watson LR Coyne-Beasley T Wood CT Cullen K. Screening for speech and language delay in children 5 years old and younger: a systematic review. Pediatrics. (2015) 136:e448–62. 10.1542/peds.2014-3889 26152671

[17] Siu AL >US Preventive Services Task Force. Screening for speech and language delay and disorders in children aged 5 years or younger: US Preventive Services Task Force Recommendation Statement. Pediatrics 136:e474–81. 10.1542/peds.2015-1711 26152670

[18] National Health and Medical Research Council (Australia). Child Health Screening and Surveillance: A Critical Review of the Evidence. (2002). Canberra: National Health and Medical Research Council.

[19] Institut für Qualität und Wirtschaftlichkeit im Gesundheitswesen. Früherkennungsuntersuchung auf umschriebene Entwicklungsstörungen des Sprechens und der Sprache: Abschlussbericht. (2009); Version 1, 0. Nr. 57. Retrieved from: https://www.iqwig.de/download/s06-01_abschlussbericht_frueherkennung_umschriebener_stoerungen_des_sprechens_und_der_sprache.pdf (accessed September 15, 2021).

[20] Kasper J Kreis J Scheibler F Möller D Skipka G Lange S. Population-based screening of children for specific speech and language impairment in Germany: a systematic review. Folia Phoniatr Logop. (2011) 63:247–63. 10.1159/000321000 21304231

[21] Uilenburg N Wiefferink K Verkerk P van Denderen M van Schie C. Accuracy of a screening tool for early identification of language impairment. J Speech Lang Hear Res. (2018) 61:104–13. 10.1044/2017_JSLHR-L-16-0173 29330554

[22] Voigt RG Llorente AM Jensen CL Fraley JK Barbaresi WJ. Comparison of the validity of direct pediatric developmental evaluation versus developmental screening by parent report. Clin Pediatr. (2007) 46:523–9. 10.1177/0009922806299100 17579105

[23] Sturrock A Marsden A Adams C. Observational and reported measures of language and pragmatics in young people with autism: a comparison of respondent data and gender profiles. J Autism Dev Disord. (2020) 50:812–30. 10.1007/s10803-019-04288-3 31758367 PMC7010622

[24] Ebert KD. Convergence between parent report and direct assessment of language and attention in culturally and linguistically diverse children. PLoS ONE. (2017) 12:e0180598. 10.1371/journal.pone.0180598 28683131 PMC5500341

[25] Miller LE Perkins KA Dai YG. (2017). Comparison of parent report and direct assessment of child skills in toddlers. Res Autism Spectr Disord. (2017) 41–42:57–65. 10.1016/j.rasd.08,002. 28919924 PMC5599144

[26] Glascoe FP. Evidence-based approach to developmental and behavioural surveillance using parents' concerns. Child Care Health Dev. (2000) 26:137–49. 10.1046/j.1365-2000.00173.x 10759753

[27] Glascoe FP. Parents' evaluation of developmental status: how well do parents' concerns identify children with behavioral and emotional problems? Clin Pediatr. (2003) 42:133–8. 10.1177/000992280304200206 12659386

[28] Glascoe FP Dworkin PH. The role of parents in the detection of developmental and behavioral problems. Pediatrics. (1995) 95:829–36. 7539122

[29] Hamann C. Specific Language Impairment in German speaking children. In: Editor Stavrakaki S, editors. Specific Language Impairment: Current trends in research. (2015). pp. 215–252. Available from: https://benjamins.com/catalog/lald.58, 10ha.m. 10.1075/lald.58.10ham

[30] Clahsen H Richman K Richman K. Child Language and Developmental Dysphasia: Linguistic Studies of the Acquisition of German. Amsterdam: John Benjamins Publishing Company. (1991)

[31] Hamann C Penner Z Lindner K. German impaired grammar: the clause structure revisited. Language Acquisition. (1998) 7:193–245. Retrieved May 27, 2021. Available from: http://www.jstor.org/stable/20000285. 10.1207/s15327817la0702-4_5

[32] Rescorla L. Late talkers: do good predictors of outcome exist? Develop Disabil Res Rev. (2011) 17:141–50. 10.1002/ddrr.1108 23362033

[33] Möller D Furche G Slabon-Lieberz S Gaumert G Breitfuss A. Blickdiagnose Sprachverständnisstörungen–Die diagnostische Güte von Experten-und Elternurteilen. Sprache· *Stimme*· *Gehör*. (2008) 32:129–35. https://10.1055/s-0028-1085435. 10.1055/s-0028-108543526606158

[34] Fenson L Marchman VA Thal DJ Dale PS Reznick JS. MacArthur-Bates communicative development inventories. 2nd edition. (2007). Baltimore: Paul H. Brookes Publishing Co. 10.1037./t11538-000

[35] Marschik PB Einspieler C Garzarolli B. Events at early development: are they associated with early word production and neurodevelopmental abilities at the preschool age? Early Hum Develop. (2007) 83, 107–14. 10.1016/j.earlhumdev.2006.05.009 16876340

[36] Grimm H Aktas M Frevert S. SETK 3-5. Sprachentwicklungstest für drei-bis fünfjährige Kinder. Diagnose von Sprachverarbeitungsfähigkeiten und auditiven Gedächtnisleistungen. (2001). Göttingen: Hogrefe.

[37] Kiese-Himmel C. AWST-R-Aktiver Wortschatztest für 3-bis 5-jährige Kinder. Göttingen: Beltz Test. (2005).

[38] Tellegen PJ Laros JA. SON-R 2 ½ - 7. Nonverbaler Intelligenztest. (2007). Göttingen: Hogrefe.

[39] Bowen DJ Kreuter M Spring B Cofta-Woerpel L Linnan L Weiner D. How we design feasibility studies. Am J Prev Med. (2009) 36:452–7. 10.1016/j.amepre.2009.02.002 19362699 PMC2859314

[40] Tomblin JB Records NL Zhang X. A system for the diagnosis of specific language impairment in kindergarten children. J Speech Hear Res. (1996) 39:1284–94. 10.1044/jshr.3906.1284 8959613

[41] Swets JA. Measuring the accuracy of diagnostic systems. Science. (1988) 240:1285–93. 10.1126/science.3287615 3287615

[42] DeLong ER DeLong DM Clarke-Pearson DL. Comparing the areas under two or more correlated receiver operating characteristic curves: a nonparametric approach. Biometrics. (1988) 44:837–45. 10.2307/2531595 3203132

[43] Pepe MS. The Statistical Evaluation of Medical Tests for Classification and prediction. Oxford University Press, New York. (2003).

[44] Jaeschke R Guyatt GH Sackett DL. Users' guides to the medical literature. III. How to use an article about a diagnostic test. B. What are the results and will they help me in caring for my patients? The Evidence-Based Medicine Working Group. JAMA. (1994) 271:703–7. 10.1001/jama.271.9.703 8309035

[45] Muthén LK Muthén B. Mplus User's Guide. (1998–2017). Los Angeles, CA: Muthén and Muthén.

[46] Robin X Turck N Hainard A Tiberti N Lisacek F Sanchez JC. pROC: an open-source package for R and S+ to analyze and compare ROC curves. BMC Bioinformatics. (2011) 12:77. 10.1186/1471-2105-12-77 21414208 PMC3068975

[47] López-Ratón M Rodríguez-Álvarez M Cadarso-Suárez C. Optimal cutpoints: an R package for selecting optimal cutpoints in diagnostic tests. J Stat Softw. (2014) 61:1–36. 10.18637/jss.v061.i08

[48] Alonzo T Pepe MS. Assessing accuracy of a continuous screening test in the presence of verification bias. J Royal Stat Soc: Series C (Appl Stat). (2005) 54:173–90. 10.1111/1460-6984.12242 26990037

[49] Hall MK Kea B Wang R. Recognising bias in studies of diagnostic tests part 1: patient selection. Emerg Med J: EMJ. (2019) 36:431–4. 10.1136/emermed-2019-208446 31302605 PMC6738935

[50] Cho H Matthews GJ Harel O. Confidence intervals for the area under the receiver operating characteristic curve in the presence of ignorable missing data. Int Stat Rev. (2019) 87:152–77. 10.1111/insr.12277 31007356 PMC6472951

[51] Enders CK Keller BT Levy R. A chained equations imputation approach for multilevel data with categorical and continuous variables. Psychol Methods. (2018) 23:298–317. 10.1037/met0000148 28557466

[52] Keller BT Enders CK. Blimp User's Manual (Version 1, 0.). (2017). Los Angeles, CA.

[53] Rubin DB. Multiple Imputation for Nonresponse in Surveys. New York, NY: Wiley. (2004).

[54] Madley-Dowd P Hughes R Tilling K. (2019). The proportion of missing data should not be used to guide decisions on multiple imputation. J Clin Epidemiol. (2019) 110:63–73. 10.1016/j.jclinepi.02,016. 30878639 PMC6547017

[55] Sim F Thompson L Marryat L Ramparsad N. Predictive validity of preschool screening tools for language and behavioural difficulties: A PRISMA systematic review. PLoS One. (2019) 14:e0211409. 10.1371/journal.pone.0211409 30716083 PMC6361441

[56] Glascoe FP. Are overreferrals on developmental screening tests really a problem?. Arch Pediatr Adolesc Med. (2001). 155, 54–9. 10.1001/archpedi.155.1.54 11177063

