# Supplementary material for: Language Screening in 3-Year-Olds: Development and Validation of a Feasible and Effective Instrument for Pediatric Primary Care
Source: Front Pediatr. 2021 Nov 23;9:752141. doi: 10.3389/fped.2021.752141 (PMC8650498; doi:10.3389/fped.2021.752141)
Supplement: Supplementary file 1 [file Data_Sheet_1.docx]

Supplementary Material

# Example illustrating the prevalence dependency of predictive values and the meaning of diagnostic likelihood ratios

Given a prevalence of 10%, a screening test with Se = .90 and Sp = .90 will result in PPV = .50 and NPV = .99. Given a prevalence of 20%, the same values for Se and Sp will result in a PPV = .69 and NPV = .97. For both scenarios, DLR+ = 9 and DLR- = .11. Formally, diagnostic likelihood ratios relate the pre-screening odds of having a disorder to the post-screening odds of having a disorder given the screening results. DLR+ is the multiplicative change in the pre-screening odds of having a LD given a positive screening result (i.e. post-screening odds = DLR+ × pre-screening odds) and DLR- is the change in the pre-screening odds of having a LD given a negative screening result (post-screening odds = DLR- × pre-screening odds). Thus, given a prevalence of 10%, the pre-screening odd of .11 (= 0.1/0.9) will change to .99 (=.11 × 9) given a positive screening result and to .01 given a negative screening result.

# Supplementary Results and Tables

Hereinafter, we present the results for the validation sample (N = 144) and to facilitate comparison also for the multiply imputed data. In short, the results for the validation sample are similar to those based on the multiply imputed data. However, as expected, sensitivity is higher and specificity is lower in the validation sample than based on multiply imputed data. This is due to verification bias. Moreover, given the higher rate of positives screens in the validation sample, PPVs in the validation sample (Table 3a) are higher than in the multiply imputed data.

## Diagnostic Accuracy of Subscales

Table 1a Diagnostic accuracy of the screening subscales for the multiply imputed data (N = 2044) and the validation sample (N = 144)

|  | *Language disorder* | |  | |  |  |  |  |
| --- | --- | --- | --- | --- | --- | --- | --- | --- |
|  | Yes | No |  |  |  | AUC-Differences - t-values (DeLong) | | |
|  | M (SD) | M (SD) | r_pb_ | AUC | 95%-CI | (1) | (2) | (3) |
| N = 2044 |  |  |  |  |  |  |  |  |
| (1) EXPRESSIVE VOCABULARY | 33.656 (17.563) | 70.810 (19.864) | -.515*** | .908 | (.864;.952) |  |  |  |
| (2) EXPRESSIVE GRAMMAR | 1.851 (2.906) | 9.165 (4.103) | -.556*** | .910 | (.859;.960) | -.039 |  |  |
| (3) SENTENCE COMPREHENSION | 5.462 (2.388) | 7.026 (1.734) | -.251*** | .705 | (.623;.786) | 4.711*** | 5.804*** |  |
| (4) NOUN PLURALS | 3.426 (4.492) | 10.056 (5.903) | -.363*** | .816 | (.745;.887) | 2.440* | 2.629* | -2.848** |
| N = 144 |  |  |  |  |  |  |  |  |
| (1) EXPRESSIVE VOCABULARY | 32.946 (19.816) | 76.358 (18.292) | -.654*** | .912 | (.866; .959) |  |  |  |
| (2) EXPRESSIVE GRAMMAR | 3.298 (3.411) | 10.742 (3.119) | -.652*** | .906 | (.856; .956) | .241 |  |  |
| (3) SENTENCE COMPREHENSION | 5.984 (2.093) | 7.577 (1.467) | -.340*** | .696 | (.594; .798) | 4.241*** | 3.550*** |  |
| (4) NOUN PLURALS | 5.390 (4.920) | 12.192 (4.982) | -.471*** | .803 | (.719; .887) | 2.439* | 2.208* | -1.832 |

*Note. PR = parent-reported, EV = expressive vocabulary, EG = expressive grammar, SC = sentence comprehension, NP = noun plurals. * p < .05, ** < .01; *** <.001. r_pb_ = point biserial correlation.*

Table 2a Logistic Regression – predicting language disorder on the basis of screening subtests for the multiply imputed data (N = 2044) and the validation sample (N = 144)

|  | Multiply imputed data (N = 2044) | | | Validation sample (N=144) | | |
| --- | --- | --- | --- | --- | --- | --- |
|  | b (SE) | Stand. b  [95%-CI] | OR | b (SE) | Stand. b  [95%-CI] | OR |
|  |  |  |  |  |  |  |
| EXPRESSIVE VOCABULARY (EV) | -.057*** (.015) | -.408 [-.579, -.237] | .945 | -.060** (.019) | -.424 [-.650, -.197] | .942 |
| EXPRESSIVE GRAMMAR (EG) | -.315*** (.066) | -.388 [-.536, -.241] | .730 | -.265** (.091) | -.372 [-.607, -.137] | .767 |
| SENTENCE COMPREHENSION (SC) | .052 (.153) | .028 [-.132, .188] | 1.053 | .024 (.162) | .013 [-.163, .190] | 1.024 |
| NOUN PLURALS (NP) | -.092 (.058) | -.160 [-.351, .031] | .912 | -.108 (.075) | -.198 [-.433,.155] | .898 |
| Threshold | -3.917 |  |  | -3.802 |  |  |
| R² | .634*** |  |  | .743*** |  |  |

*Note. OR = odds ratio, * p < .05, ** < .01; *** <.001.*

## Diagnostic accuracy of the composite screening score

In the validation sample, the AUC for the composite score was also excellent at .938 (DeLong 95% CI = [.884, .991]. DeLong tests for paired ROC curves indicate that the composite shows higher AUC-values than the single parent reported scales. However, only the difference between the screening composite (COMP) and expressive grammar was significant (COMP vs. Expressive Vocabulary: ΔAUC = .026, t-value = 1.575, p > .05; COMP vs. Expressive Grammar: ΔAUC = .032, t-value = 2.288, p = .022).

## Cut-off estimation

In the validation sample, the “SpEqualSe” criterion of the Optimal Cutoff Package yielded a cut-off at 35.80 that was most efficient and resulted in satisfactory accuracy statistics: sensitivity = .825 (95%-CI = [.718, .932]), specificity = .842 (95%-CI = [.887, .897]), PPV = .668 (95%-CI = [.532, .803]), NPV = .926 (95%-CI = [.873, .979]), DLR+ = 5.232 (95%-CI = [3.023, 7.440]), DLR- = .208 (95%-CI = [.074, .342]).

Table 3a Diagnostic accuracy statistics for various cut-offs for the multiply imputed data (N = 2044) and the validation sample (N = 144)

| Cutoff | %-Screening  positives | Sensitivity | Specificity | PPV | NPV | DLR+ | DLR- |
| --- | --- | --- | --- | --- | --- | --- | --- |
| **Multiply imputed data (N = 2044)** | | |  |  |  |  |  |
| 35 | .092 | .609 (.475, .742) | .965 (.954,.977) | .658 (.538, .779) | .956 (.929, .983) | 17.813 (10.418, 25.187) | .406 (.268, .543) |
| 36 | .111 | .661 (.532, .790) | .950 (.936, .963) | .591 (.470, .713) | .961 (.936, .986) | 13.294 (8.735, 17.843) | .357 (.211, .493) |
| 37 | .124 | .697 (.568, .828) | .939 (.924, .954) | .557 (.438, .677) | .965 (.941, .988) | 11.518 (7.936, 15.100) | .323 (.187, .460) |
| 38 | .138 | .742 (.615, .868) | .929 (.913, .945) | .536 (.418, .654) | .969 (.947, .992( | 10.547 (7.545, .13.548) | .278 (.143, .413) |
| 39 | .150 | .779 (.655, .903) | .919 (.902, .936) | .516 (.399, .632) | .973 (.952, .995) | 9.704 (7.156, 12.252) | .240 (.106, .375) |
| 40 | .169 | .820 (.701, .939) | .904 (.886, .922) | .485 (.374, .596) | .978 (.958, .997) | 8.553 (6.620, .10.480) | .199 (.068, .330) |
| 41 | .189 | .865 (.755, .976) | .886 (.867, .905) | .456 (.348, .564) | .983 (.965, 1.000) | 7.612 (6.127, 9.907) | .152 (.028, .276) |
| 42 | .202 | .879 (.773, .985) | .873 (.853, .892) | .433 (.328, .538) | .984 (.967, 1.000) | 6.918 (5.678, 8.159) | .139 (.018, .259) |
|  |  |  |  |  |  |  |  |
| **Validation sample (N = 144)** | | |  |  |  |  |  |
| 35 | .314 | .800 (.689, .911) | .873 (.822, .924) | .708 (.574, .841) | .919 (.865, .973) | 6.298 (3.314, 9.283) | .229 (.097, .362) |
| 36 | .350 | .827 (.719, .934) | .833 (.779, .887) | .656 (.524, .787) | .926 (.873, .979) | 4.949 (2.994, 6.904) | .208 (.073, .343) |
| 37 | .374 | .875 (.779, .971) | .819 (.764, .875) | .651 (.522, .780) | .945 (.897, .992) | 4.851 (3.099, 6.593) | .153 (.032, .274) |
| 38 | .393 | .925 (.847, 1.000) | .812 (.757, .867) | .654 (.5390, .779) | .966 (.928, 1.000) | 4.921 (3.303, 6.538) | .092 (.000, .191) |
| 39 | .408 | .925 (.847, 1.000) | .790 (.734, .846) | .629 (.505, .754) | .965 (.926, .1.000) | 4.417 (3.076, 5.754) | .095 (.000, .196) |
| 40 | .426 | .950 (.884, 1.000) | .776 (.720, .833) | .620 (.498, .743) | .976 (.943, 1.000) | 4.246 (3.067, 5.426) | .064 (.000, .150) |
| 41 | .453 | .950 (.884, 1.000) | .738 (.682, .795) | .583 (.463, .703) | .975 (.940, 1.000) | 3.634 (2.751, .4.515) | .068 (.000, .158) |
| 42 | .481 | .950 (.884, 1.000) | .699 (.644, .754) | .548 (.431,.666) | .973 (.937,1.000) | 3.156 (2.477, 3.833) | .072 (.000, .168) |

*Note.95%-CI in brackets.*
